# Impaired Expression of Chloroplast HSP90C Chaperone Activates Plant Defense Responses with a Possible Link to a Disease-Symptom-Like Phenotype

**DOI:** 10.3390/ijms21124202

**Published:** 2020-06-12

**Authors:** Shaikhul Islam, Sachin Ashok Bhor, Keisuke Tanaka, Hikaru Sakamoto, Takashi Yaeno, Hidetaka Kaya, Kappei Kobayashi

**Affiliations:** 1 The United Graduate School of Agricultural Sciences, Ehime University, Matsuyama, Ehime 790-8566, Japan; islamshaikhul2014@outlook.com (S.I.); bhor.sach@gmail.com (S.A.B.); yaeno@agr.ehime-u.ac.jp (T.Y.); kaya.hidetaka.hu@ehime-u.ac.jp (H.K.); 2 NODAI Genome Research Center, Tokyo University of Agriculture, Setagaya, Tokyo 156-8502, Japan; kt205453@nodai.ac.jp; 3 Faculty of Bio-Industry, Tokyo University of Agriculture, Abashiri, Hokkaido 099-2493, Japan; h3sakamo@nodai.ac.jp; 4 Graduate School of Agriculture, Ehime University, Matsuyama, Ehime 790-8566, Japan; 5 Research Unit for Citromics, Ehime University, Matsuyama, Ehime 790-8566, Japan

**Keywords:** cell death, chlorosis, HSP90C, immune response, RNA-seq, tobacco, transcriptome

## Abstract

RNA-seq analysis of a transgenic tobacco plant, i-hpHSP90C, in which chloroplast HSP90C genes can be silenced in an artificially inducible manner resulting in the development of chlorosis, revealed the up- and downregulation of 2746 and 3490 genes, respectively. Gene ontology analysis of these differentially expressed genes indicated the upregulation of ROS-responsive genes; the activation of the innate immunity and cell death pathways; and the downregulation of genes involved in photosynthesis, plastid organization, and cell cycle. Cell death was confirmed by trypan blue staining and electrolyte leakage assay, and the H_2_O_2_ production was confirmed by diaminobenzidine staining. The results collectively suggest that the reduced levels of HSP90C chaperone lead the plant to develop chlorosis primarily through the global downregulation of chloroplast- and photosynthesis-related genes and additionally through the light-dependent production of ROS, followed by the activation of immune responses, including cell death.

## 1. Introduction

Plant virus diseases develop a range of symptoms resulting from the morphological and physiological disturbances of the host cells. Leaf chlorosis, the most frequently observed symptom, reduces plant productivity and thus leads to a significant loss in crop yield. Virus-induced chlorosis often accompanies the structural change and dysfunction of chloroplasts, including the reduction in chlorophyll content and the expression of photosynthetic genes [1,2,3,4]. Therefore, understanding the mechanisms of chloroplast dysfunction would lead us to the establishment of countermeasures against crop loss. Although the molecular events during chlorosis have been extensively documented, the precise mechanism for the reduced chloroplast activities had remained to be elucidated until recent pioneering studies showed the involvement of RNA silencing of chloroplast protein genes in the development of chlorosis by subviral RNAs. Two groups have independently shown that the bright yellow symptoms in tobacco plants infected with cucumber mosaic virus (CMV) harboring Y-satellite RNA (Y-sat) is attributed to the RNA silencing of magnesium protoporphyrin chelatase subunit I (CHLI) involved in chlorophyll biosynthesis mediated by the Y-sat-derived small interfering RNA (siRNA) [5,6]. Another study has shown that siRNA derived from *peach latent mosaic viroid* (PLMVd) directs the RNA silencing of chloroplast heat-shock protein 90 (HSP90C) and consequently causes severe chlorosis or albinism in peach trees, which are the natural hosts of the viroid [7]. A recent study identified an additional pathogenic determinant of yellow mosaic in peach, PLMVd-sRNA40, which guides cleavage of the mRNA encoding a thylakoid translocase subunit required for chloroplast development [8].

To analyze the molecular mechanisms of chlorosis induced by the RNA silencing of chloroplast proteins, we previously established experimental systems using a chemically inducible promoter to drive RNAs to induce RNA silencing of CHLI and HSP90C in transgenic tobacco plants [9,10]. We believe such systems have an advantage over the artificial infection of natural or experimental hosts with pathogens. Firstly, they facilitate the analysis of early host response to the pathogenic determinants because it is possible to analyze plant tissue committed to developing chlorosis but not yet exhibiting visible chlorosis. Secondly, they facilitate the analysis of temporal changes in host response because cells in the model plants are expected to undergo molecular changes in a near-synchronous manner. Finally, they promote the discovery of some hidden host responses because the current plant–pathogen interactions have been shaped through the multilayered evolution of hosts’ defense and pathogens’ counter-defense [11,12]. Therefore, the analyses in the inducible model plants with isolated pathogenic triggers can promote our understanding of the precise mechanisms underlying the pathogenesis in plant infectious diseases.

In plants, the HSP90 family comprises 7, 9, and 10 members in *Arabidopsis thaliana*, *Oryza sativa*, and *Populus trichocarpa*, respectively [13,14]. Out of seven *Arabidopsis* HSP90 family proteins, four members exhibit nucleo-cytoplasmic localization, while the other three localize to the chloroplast, mitochondrion, or endoplasmic reticulum (ER) [13]. The HSP90C or AtHSP90.5, the protein product of which localizes to the chloroplast, was first identified as the causal gene for a chlorate-resistant mutation [15]. Like cognate proteins in the mitochondrion [16,17] or ER [18,19], HSP90C has been proposed to have a role in protein folding in the chloroplast [20] and has shown to be essential for the transport of different proteins into chloroplasts [21]. Although the chlorate-resistant mutant, cr88, which has a single missense mutation in the HSP90C coding sequence, was viable, albeit with an impaired photomorphogenesis [15], null mutants with T-DNA insertions in the HSP90C gene were lethal [21,22], indicating its essential function. The silencing of HSP90C in Arabidopsis plants resulted in variegated or albino phenotypes [23]. Therefore, the finding that the silencing of HSP90C by viroid-derived siRNA resulted in severe chlorosis or albinism was in line with the phenotype observed in the mutants [7].

We previously showed that the induced silencing of HSP90C in transgenic tobacco resulted not only in visible chlorosis with significantly decreased chlorophyll contents and the reduced expression of chloroplast protein genes but also in the induction of some pathogenesis-related genes [10]. The results suggest that the chlorosis in this model system is attributed not only to the impaired chloroplast biogenesis but also to active plant responses to the impaired chloroplast function. In this report, we employed an RNA-seq analysis to examine the alteration in gene expression shortly after the induction of HSP90C silencing. By comparing the gene expression patterns between HSP90C silenced and non-silenced plants, we found upregulation of genes related to the response to reactive oxygen species, cell death, plant hormone signaling pathways, defense response, and innate immune response. The detection of sporadic cell death supported the biological significance of the transcriptomic changes. The results suggest that chlorosis development with impaired HSP90C expression involves the activation of cell-death-mediated plant defense response in addition to a simple reduction of chloroplast function.

## 2. Results

### 2.1. RNA Sequencing, Mapping, and Identification of Differentially Expressed Genes (DEGs)

**Figure 1:**
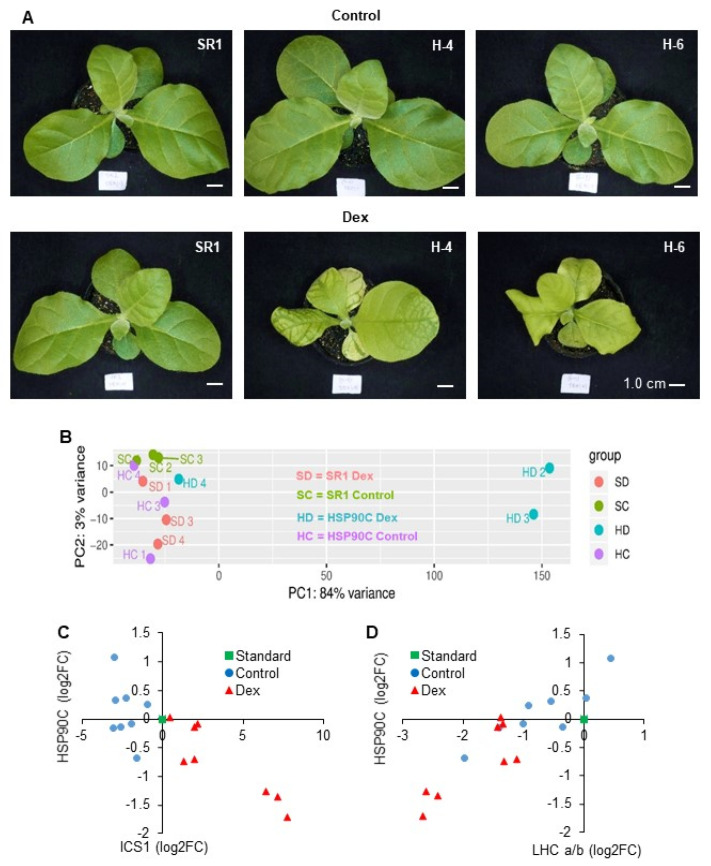
Phenotype and transcriptome change after induced silencing of HPS90 in transgenic tobacco plants. (**A**) Non-transformed SR1 (SR1), i-hpHSP90C 4–5 (H-4), and i-hpHSP90C 6-1 (H-6) plants were grown for three weeks, sprayed with 0.01% Tween-20 containing 0.5% ethanol (Control) or 50 µM Dex (Dex), and photographed at 7 days post-treatment (dpt). Scale bars indicate 1 cm. (**B**) Principle component analysis of RNA-seq data. RNA preparations from 1-dpt control-treated SR1 (SC2, 3, and 4), Dex-treated SR1 (SD1, 3, and 4), control-treated i-hpHSP90C-4 (HC1, 3, and 4), and Dex-treated i-hpHSP90C-4 (HD2, 3, and 4) were analyzed for the differential gene expression by using the DESeq2 package in Galaxy platform. (**C**,**D**) Variation of HSP90C silencing in individual Dex-treated i-hpHSP90C-4 plants. Expression levels of ICS1 (**C**) and LHC a/b (**D**), representatives of up- and downregulated genes found in the RNA-seq analysis, respectively, were plotted with those of HSP90C. The expression levels of these genes relative to a fixed standard sample (square) were quantified in eight individual plants from each of the control- (circles) and Dex-treated (triangles) i-hpHSP90C-4 plant groups.

We previously reported that the silencing of HSP90C in transgenic tobacco lines resulted in chlorosis and growth suppression (Figure 1A) accompanied by an activation of pathogenesis-related (PR) genes [10]. RNA-seq analysis was conducted to elucidate the mechanisms underlying the development of chlorosis in this model system. Three-week-old i-hpHSP90C transgenic line H-4 and non-transformant SR1 were Dex- or control-treated, and RNA was extracted at 24 h post-Dex-treatment from four individual plants from each plant/treatment group. Three out of each group of four plants were selected for RNA-seq based on their integrity and purity (data not shown). The RNA sequencing gave 23 M reads/sample on average from the 12 samples, 80–90% of which were mapped to the tobacco reference transcriptome (Supplementary Table S3).

**Figure 2:**
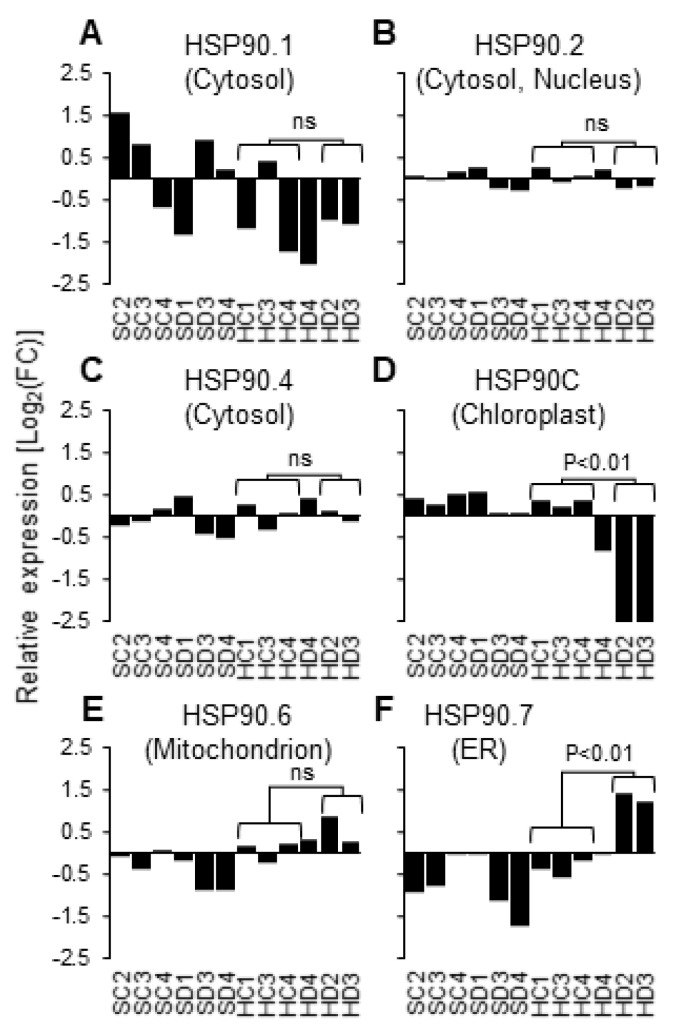
Expression changes of HSP90 family genes in RNA-seq samples. Relative normalized read counts in all RNA-seq samples are shown for tobacco homologs of Arabidopsis HSP90 family genes: (**A**) cytosolic HSP90.1; (**B**) nucleo-cytosolic HSP90.2; (**C**) cytosolic HSP90.4; (**D**) chloroplast HSP90C or HSP90.5; (**E**) mitochondrial HSP90.6; (**F**) ER-localized HSP90.7. Statistical significance of the difference in read counts was evaluated between control-treated (HC1, 3, and 4) and Dex-treated (HD2 and 3) in DESeq2 analysis. ns, not significant. Data from the HD4 sample were omitted as mentioned in the text.

In our initial analysis, the overall similarity within samples was evaluated by a principal component analysis (PCA) (Figure 1B). The result showed that two samples, HD2 and HD3, showed clear differences from the control samples including the non-transformed SR1 samples regardless of the treatment. In contrast, one of the Dex-treated i-hpHSP90C plants (HD4) showed the least differences from those control samples (Figure 1B). The higher normalized HSP90C transcript counts indicated that the HD4 plant had not efficiently been silenced in HSP90C expression (Figure 2D), suggesting that the transcriptomic changes are observed only after a significant HSP90C downregulation. To test the hypothesis, we compared the expression levels by qRT-PCR of HSP90C and representative nuclear genes for chloroplast proteins that show clear up- and downregulation found in the RNA-seq analysis (see below) for the isochorismate synthase 1 gene (ICS1) and a light-harvesting chlorophyll a/b-binding protein (LHC a/b), respectively. The gene expression levels of ICS1 and LHC a/b correlated with the HSP90C expression negatively and positively, respectively (Figure 1C,D). Importantly, in some plants of the i-hpHSP90C H-4 line, in which HSP90C expression levels were more than half those of the control plants, the upregulation of ICS1 and the downregulation of LHC a/b were not very prominent and were within the variability of control (Dex-untreated) plants. In contrast to the H-4 line, the H-6 line of i-hpHSP90C transgenic plants showed highly reproducible HSP90C silencing and the up- and downregulation of ICS1 and LHC a/b, respectively (Supplementary Figure S1). The SR1 plant did not respond to Dex treatment in the expression of the genes examined. These results support the hypothesis above; therefore, we omitted the HD4 sample from the differential expression analysis.

**Figure 3:**
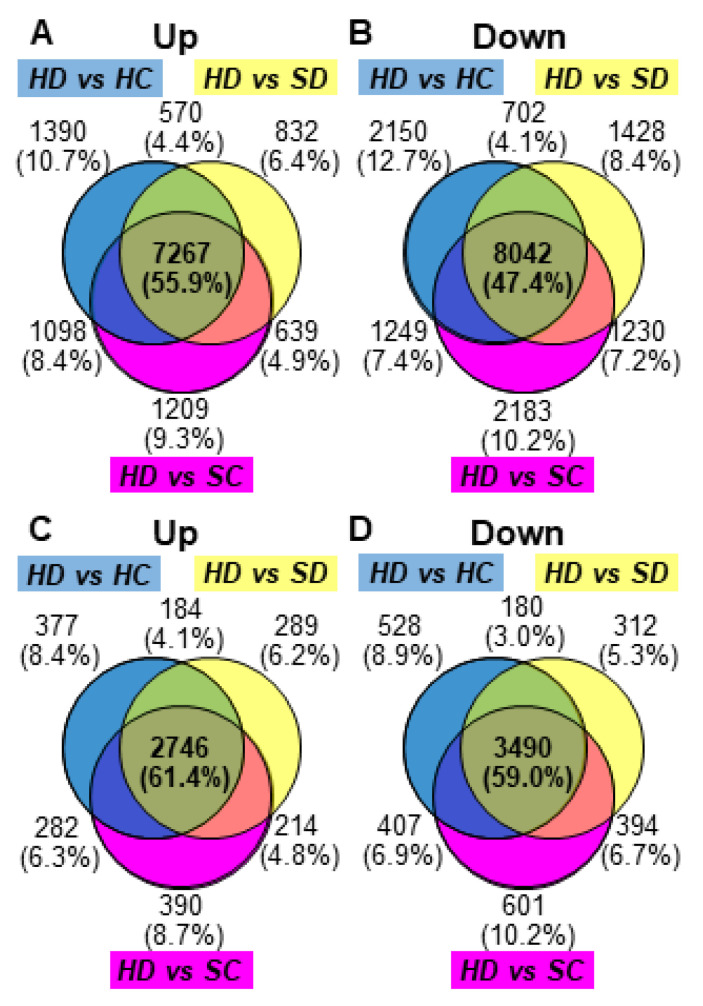
Venn diagrams of differentially expressed genes. Results from differential expression analysis with DESeq2 were considered in three different comparisons: Dex-treated H-4 vs. control H-4 (HD vs. HC), Dex-treated H-4 vs. Dex-treated SR1 (HD vs. SD), and Dex-treated H-4 vs. Control SR1 (HD vs. SC). The differentially expressed genes (DEGs) were detected using DESeq2 as mentioned in Section 4. Diagrams (**A**) and (**B**) show DEGs detected in the analysis with mRNA ID in the reference transcriptome, and diagrams (**C**) and (**D**) show those annotated with AGI codes. Diagrams (**A)** and (**C**) show numbers of upregulated genes, whereas diagrams (**B**) and (**D**) show those of downregulated genes.

The gene expression of HD2 and HD3 samples (HD) were compared using DESeq2 with all three groups, untreated line H-4 (HC), Dex-treated SR1 (SD), and untreated SR1 (SC), which have never shown chlorosis in our repeated experiments. DEGs were picked up from the DESeq2 data based on the combined criteria of log2(FC) values below −1 or above 1 and adjusted p-values less than 0.05. The MA plots support that the three comparisons identified a consistent set of DEGs (Supplementary Figure S2). The analyses of differentially expressed mRNA transcripts in the three different comparisons above—HD vs. HC, HD vs. SD, and HD vs. SC—identified 7267 (55.9%) and 8042 (47.4%) commonly up- and downregulated mRNAs, respectively (Figure 3A,B). Because the mRNA IDs in the tobacco reference transcriptome are not readily used for downstream GO enrichment analysis, those annotated with the Arabidopsis AGI codes were selected. Out of the differentially expressed mRNAs above, 4896 upregulated mRNAs had annotation with 2746 different AGI codes comprising 61.4% of DEGs with AGI annotation common in three comparisons, and 6307 downregulated mRNAs had 3490 (59.0%) AGI codes (Figure 3C,D and Supplementary Tables S4 and S5).

### 2.2. Functional Classification of DEGs

**Figure 4:**
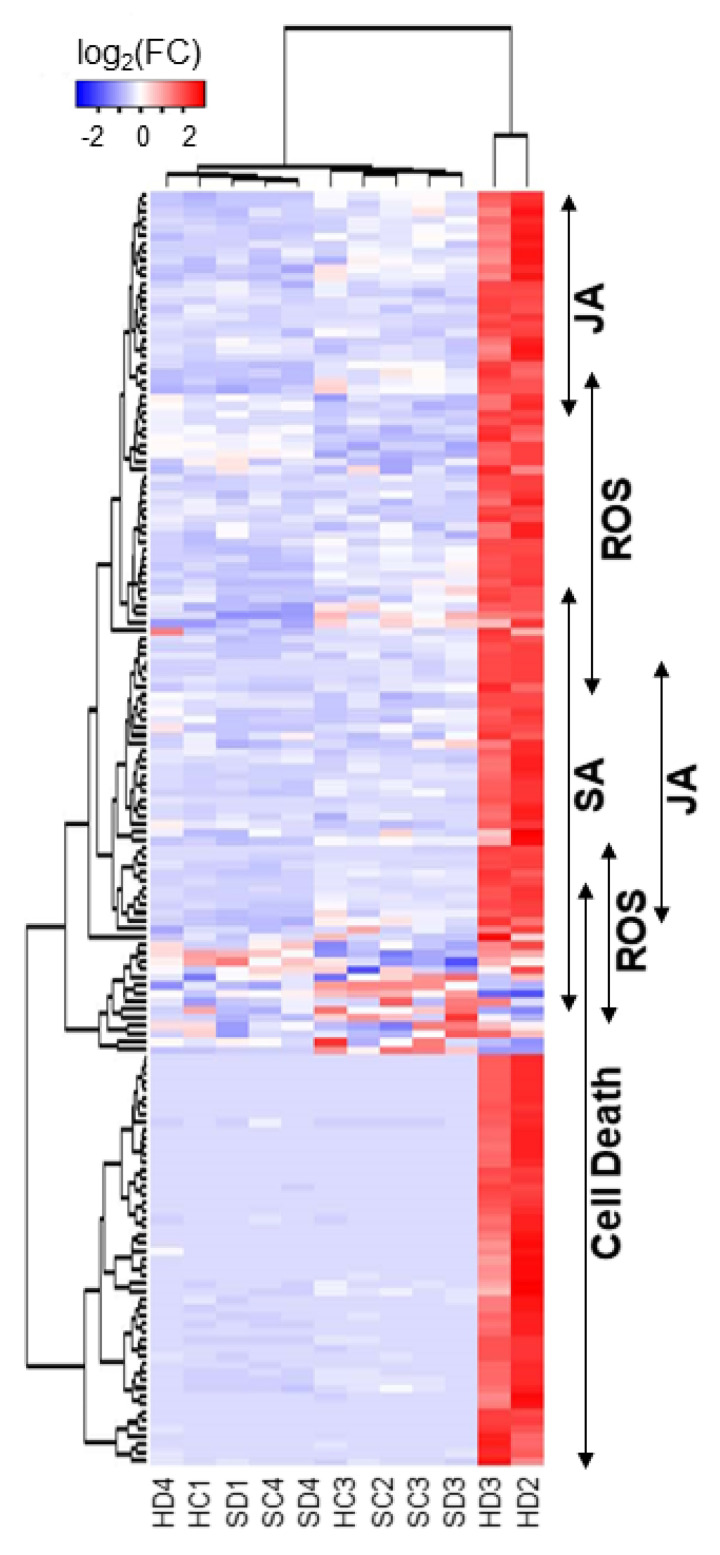
Hierarchical clustering of upregulated DEGs with selected annotations. The genes annotated with GO terms “response to SA” (GO:0009751), “response to JA” (GO:0009753), “response to oxidative stress” (GO:0006979), and “cell death” (GO:0008219) were selected (Supplementary Table S9), and their relative normalized count data were used to draw a heatmap. Each column represents a sample, and each row represents a selected gene. Differences in expression are shown in different colors, where red and blue represent up- and downregulated expression, respectively.

The AGI codes given to the up- and downregulated mRNAs were used for the GO enrichment analysis. Table 1 and Table 2 present the selected GO terms for biological processes enriched in the lists of up- and downregulated mRNAs, respectively. The enriched GO terms in the upregulated genes include “innate immune response”, “response to wounding”, “response to oxidative stress”, “response to phytohormones” (salicylic acid (SA), jasmonic acid (JA), and abscisic acid (ABA)), and “hypersensitive cell death” (Table 1 and Supplementary Table S6). In accordance with the observations, biosynthetic genes of SA and JA were found upregulated (Table 1). The heatmap (Figure 4) shows the expression changes of genes selected from those in GO terms “response to SA” (GO:0009751), “response to JA” (GO:0009753), “response to oxidative stress” (GO:0006979) and “cell death” (GO:0008219), which are indicated by double-headed arrows on the right. The results suggest that the reduced supply of HSP90C to chloroplast elicits defense response involving some phytohormone pathways.

In addition to the activation of defense response, genes involved in the response to ER stress were remarkably upregulated after the impaired supply of HSP90C (Table 1). This observation could be attributed to the non-specific silencing of ER-localizing HSP90 family protein by the HSP90C hairpin RNA (hpRNA). Therefore, we examined the expression levels of six out of seven HSP90 family proteins in the RNA-seq data because no tobacco mRNA in the reference transcriptome was annotated with AT5G56010 encoding a nucleo-cytosolic HSP90.3. The true target of hpRNA-mediated silencing, HSP90C or HSP90.5, was significantly downregulated, as shown in Figure 2D, but the expression levels of cytosolic HSP90.1 and HSP90.4, nucleo-cytosolic HSP90.2, and mitochondrial HSP90.6 were not affected by the Dex treatment (Figure 2A–C,E). In contrast, statistically significant upregulation in Dex-treated HD2 and HD3 was detected in ER-localizing HSP90.7 (Figure 2F). The results contradict the off-target silencing of ER-localizing HSP90. One may assume a possible interplay between the protein quality control system of ER with that in plastid, although the ER-localizing HSP90 and other ER protein quality control components could have been upregulated through the activation of SA production or immune responses [24,25].

The enriched GO terms (biological process) in the downregulated genes include “photosynthesis”, “pigment metabolic process”, and “plastid organization” (Table 2 and Supplementary Table S7), which is consistent with crucial roles of HS90C in chloroplast biogenesis. In addition, some primary metabolism genes annotated with the “carbohydrate metabolic process”, “lipid metabolic process” and “cellular amino acid metabolic process” GO terms; other metabolic genes annotated with “cofactor metabolic process” and “vitamin metabolic process” GO terms; and some cellular process genes annotated with “cell cycle”, “cell wall organization”, and “cellular homeostasis” GO terms were shown to be downregulated (Table 2 and Supplementary Table S7), which is consistent with the upregulation of cell-death-related genes (Table 1 and Supplementary Table S6). Interestingly, three GO terms, namely “response to osmotic stress”, “response to oxidative stress”, and “response to salt stress”, were enriched in the downregulated genes. However, they were also enriched in the upregulated genes (Table 1 and Table 2). The results suggest that impaired HSP90C supply would induce drastic switching in these stress-responsive genes (Supplementary Figure S3).

### 2.3. Detection of Cell Death and Reactive Oxygen Species in HPS90C-Silenced Plants

**Figure 5:**
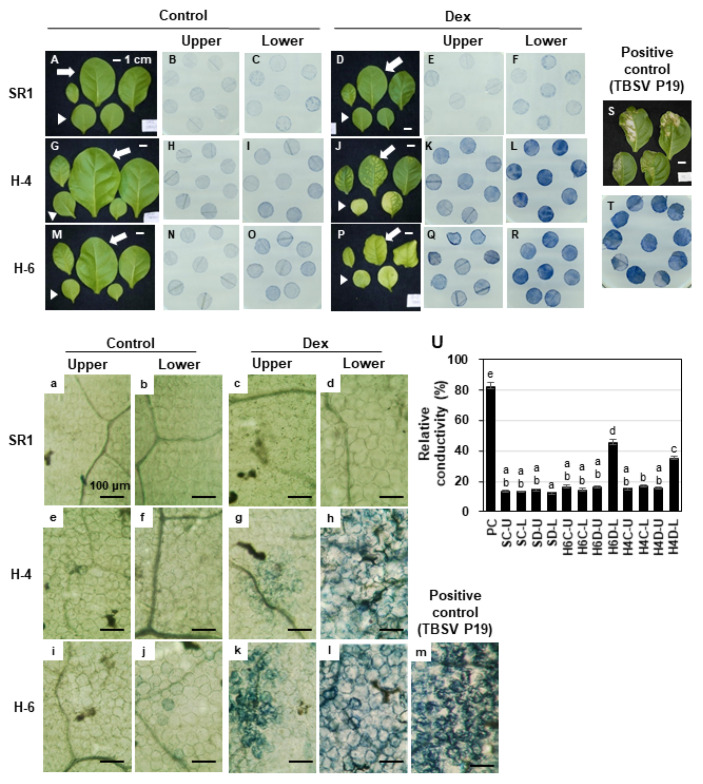
Detection of cell death in chlorotic leaves. Control plants (SR1; **A**–**F** and **a**–**d**) and i-hpHSP90C transgenic line 4 (H-4; **G**–**L** and **e**–**h**) and line 6 (H-6; **M**–**R** and **i**–**l**) plants were grown and treated with control or Dex solution. They were harvested and photographed at 7-dpt (**A**,**D**,**G**,**J**,**M**,**P**,**S**; representative plants from triplicate experiments; scale bars denote 1 cm) followed by cell death assays. SR1 leaves transiently expressing TBSV P19 for 2 days served as a positive control (**S**,**T**,**m**). Cell death assay was performed in upper leaves (indicated by arrows) and lower leaves (indicated by arrowheads) Leaf disks of 6 mm in diameter were stained with trypan blue and photographed at macroscopic (**B**,**C**,**E**,**F**,**H**,**I**,**K**,**L**,**N**,**O**,**Q**,**R**,**T**) and microscopic (**a**–**m**; scale bars denote 100 μm) levels. (**U**), quantification of cell death by an electrolyte leakage assay; PC, positive control with transient TBSV P19 expression; SC, control-treated SR1; SD, Dex-treated SR1; H4C, control-treated line 4; H4D, Dex-treated line 4; H6C, control-treated line 6; H6D, Dex-treated line 6; U, upper leaves; L, lower leaves. Error bars denote standard deviations in triplicate experiments. Different letters indicate statistically significant differences between treatments (LSD test, *p* < 0.05). The experiment was repeated at least three times.

The significant upregulation of the cell death pathway prompted us to detect cell death in i-hpHSP90C lines. Because lower (older) leaves showed more severe chlorosis than the upper (younger) leaves, we examined the cell death in those leaves separately. The trypan blue staining showed light blue staining in upper leaves and more intense staining in older leaves of Dex-treated H-4 and H-6 plants, albeit to a lesser extent than the positive control (Figure 5J–L,P–R). In the positive control plants, in which TBSV P19 had transiently been expressed for two days, some leaf discs were cut out to include both dead and living parts to make the difference in staining of those parts clear (Figure 5S,T). The staining of lower leaves from Dex-treated H-4 and H-6 plants was not uniform, suggesting the cell death induction in those leaves was sporadic within the leaf tissue. Electrolyte leakage assay confirmed the cell death in lower leaves but not in upper leaves (Figure 5U). Microscopic observation of trypan-blue-stained leaf tissue indicated that dead cells in lower leaves of Dex-treated H-4 and H-6 plants were barely shrunken (Figure 5h,l), unlike those in the positive control (Figure 5m). The upper leaves exhibited small patches of dead cells, suggesting the sporadic and age-dependent natures of cell death in Dex-treated H-4 and H-6 plants (Figure 5g,k). A small fraction of cells in lower but not upper leaves of untreated H-6 showed blue staining (Figure 5j), suggesting that the hpRNA to HSP90C had been expressed in a leaky fashion in a small number of cells in the untreated H-6 line and that the cell death observed is age-dependent. Dex-treated and untreated control plants (SR1) and untreated H-4 did not show any signs of cell death (Figure 5a–f). These results collectively suggest that the death of cells, in which HSP90C supply is impaired, is stochastically initiated and proceeds somewhat slowly, although the RNA-seq suggests that it is a plant-type hypersensitive response (Table 1).

**Figure 6:**
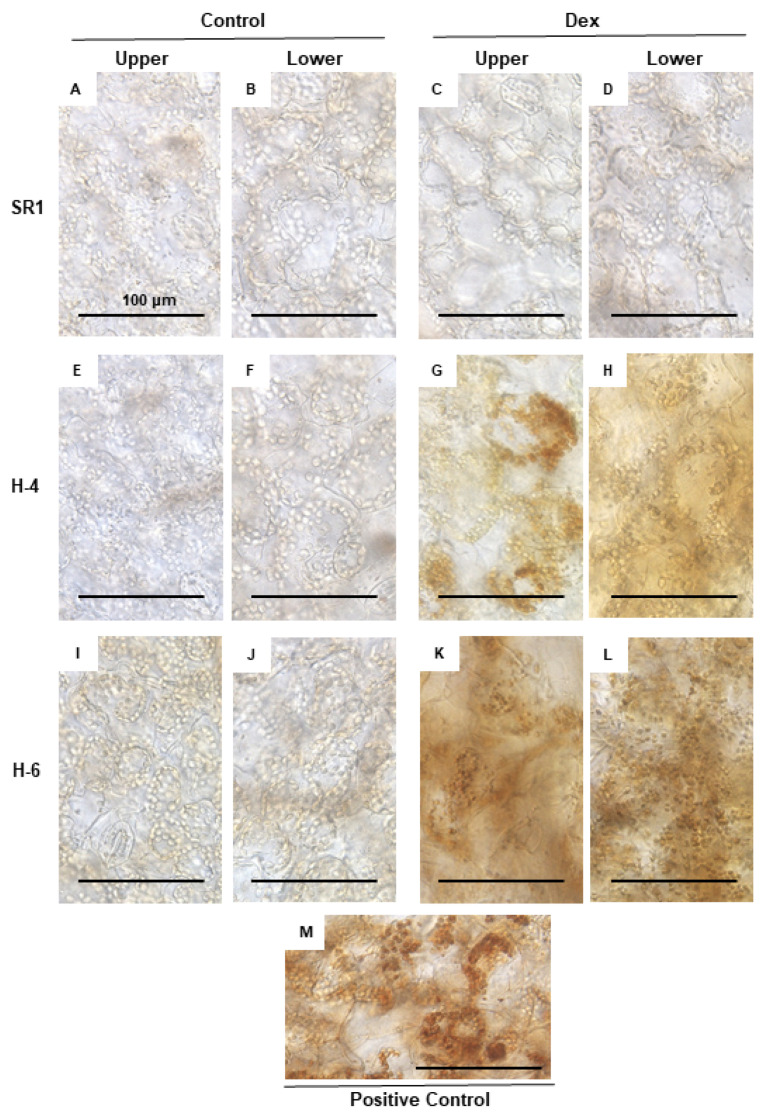
H_2_O_2_ production in the chloroplasts of leaves developing chlorosis. Control plants (SR1; **A**–**D**), i-hpHSP90C transgenic line 4 (H-4; **E**–**H**), and line 6 (H-6; **I**–**L**) plants were grown and treated with control or Dex solution. At 24 h post-treatment, leaf disks of 6 mm in diameter were cut out from upper and lower leaves, vacuum-infiltrated with DAB solution, and incubated under light conditions (70–100 µmol m^−2^ s^−1^ for 30 min). Untreated SR1 plants illuminated with 250–300 µmol m^−2^ s^−1^ for 60 min served as a positive control (**M**). All leaf disks were incubated in the dark for 3.5 h, decolorized, and observed under a microscope.

The upregulation of genes involved in the response to oxidative stress (Table 1 and Figure 4) suggests the production of reactive oxygen species (ROS) in Dex-treated H-4 and H-6 plants. Because ROS is well studied as a signal mediator and an executor of plant cell death, we tried to detect H_2_O_2_ as a representative of ROS. Positive control with intense light resulted in the accumulation of brownish DAB precipitate in chloroplasts (Figure 6M). Intense DAB staining of chloroplasts was barely visible in Dex-treated and untreated control plants (SR1) and in untreated H-4 and H-6 plants (Figure 6A–F,I,J). Evident DAB staining of chloroplasts was observed in leaves of Dex-treated H-4 and H-6 plants at 1-dpt (Figure 6G,H,K,L), but it was not observed when cell death was observed at 7-dpt (data not shown). The results suggest that, upon the loss of sufficient levels of HSP90C, chloroplasts produce ROS, triggering the cell death response. Although the overall DAB pigmentation was more intense in lower leaves, especially in H-4, pigmentation of each chloroplast was comparable between upper and lower leaves. The observation suggests that the extents of cell death correlate with the number of cells producing ROS but not with the magnitude of ROS production.

## 3. Discussion

We previously established an inducible silencing system for HSP90C in tobacco and confirmed that the silencing of HSP90C alone could lead plants to chlorosis [10]. The system has an advantage over the experimental systems with the virus- or viroid-infected plants in the analysis of mechanisms underlying the development of disease-symptom-like phenotypes such as chlorosis. It is possible in this system to analyze plant cells that have committed to developing chlorosis but are not yet exhibiting visible chlorosis. Exploiting this advantage in the present study, we explored the early molecular changes leading to the development of chlorosis using RNA-seq analysis. Although studies have shown the transcriptome changes in virus- and viroid-infected plants [3,26,27], the strength of the present study is that we could detect any changes that precede detectable chlorosis.

From two independent transgenic lines, H-4 and H-6, which show mild and severe chlorosis and growth suppression after Dex treatment, respectively, we selected the former for the RNA-seq analysis. In the triplicate experiment, an individual Dex-treated H-4 plant (HD4 in Figure 1B and Figure 2) showed a moderate reduction in HSP90C expression levels (Figure 2D) and the lack of transcriptome changes observed in two other Dex-treated H4 plants (Figure 1B and Figure 4 and Supplementary Figure S3). Because such a variation within individual Dex-treated H-4 plants was confirmed by qRT-PCR (Figure 1C,D), we omitted the HD4 data from our RNA-seq data analysis. Although more biological replicates are recommended for reliable RNA-seq data analysis in general, the DESeq2 used in this study has been shown to give the lowest false positive rate [28]. Therefore, most of the data analysis results were taken into account in this report. In contrast to Dex-treated H-4 plants, Dex-treated H-6 plants showed drastic downregulation of LHC a/b expression and dramatic induction of ICS1 expression. The results suggest a more rapid progression of molecular changes toward chlorosis in H-6 than in H-4 plants, which may be more suitable than H-6 for analyzing the early molecular changes.

We found several characteristic transcriptome changes in HD2 and HD3 plants, which we believed to be on the way to develop chlorosis. Firstly, downregulation was observed in genes involved in (group 1) photosynthesis and plastid organization, (group 2) primary and secondary metabolisms, and (group 3) cell and plant growth. Secondly, the genes involved in (group 4) immune response accompanying cell death and (group 5) ER stress response were found to be upregulated. Finally, the genes involved in response to different abiotic stresses showed a mixed response, with one subset being upregulated and another subset being downregulated.

Among the transcriptome changes above, downregulation of chloroplast- and photosynthesis-related genes (CPRGs) or group 1 genes has been widely reported in symptomatic tissues of different combinations of host plants and viruses [3,29,30,31]. Although it is natural that the expression of CPRGs is downregulated in chlorotic tissue, the present study, together with our previous results [9,10,32,33], strongly suggests that the downregulation of CPRGs preceding visible chlorosis is the primary pathway of chlorosis development. The mechanism underlying the downregulation of CPRGs may differ within pathosystems, but the present study suggests a possible involvement of retrograde signaling (RS) in chlorosis development induced by subviral pathogens. The downregulation of genes in groups 2 and 3 can also be attributed to the RS activation, which reprograms transcriptomes from the growth and differentiation state to the stress response state [34]. The RNA-seq data suggest the activation of RS pathways as manifested by the increased expression of transcription factors involved in RS-mediated transcriptome changes and decreased expression of those involved in chloroplast biogenesis (Supplementary Table S8) [35]. Given that RS pathways are activated in the chlorosis model of the present study, ROS could be a primary signal, as we detected H_2_O_2_ production in chloroplasts the day after the induction of HSP90C silencing. H_2_O_2_ molecules are assumed to move across the biological membrane [36] and thus would move from the chloroplast to the nucleo-cytoplasmic space to activate different signaling pathways. Although transcriptome profile and ROS detection support the RS activation during chlorosis development, other RS signaling molecules should be analyzed to confirm the activation of RS pathways in the present model system. It is noteworthy that cyanobacterial HSP90, HtpG, interacts with and modulates the activity of uroporphyrinogen decarboxylase and thus regulates tetrapyrrole biosynthesis [37].

Among group 4 or immunity genes, ICS1 was highly upregulated in the chlorosis model (Figure 1C, Supplementary Figure S1, and Supplementary Tables S4 and S6). In addition to pathogen attack and UV irradiation, ICS1 expression is reportedly upregulated with excess light and β-cyclocitral, a retrograde signaling molecule produced by the oxidation of β-carotene with singlet oxygen [38]. The upregulation of ICS1 expression in the present model system could also be regulated by some signals from the chloroplast to the nucleus. Our RNA-seq data has indicated significant upregulation of three transcription factors, namely SARD1, CBP60g, and WRKY28, which have significant roles in ICS1 gene activation [39,40,41,42]; their flg22-induced expression reportedly depends on the chloroplast-localized calcium sensor (CAS) [43]. Because cytoplasmic calcium-dependent protein kinases have been shown to regulate those transcription factors [44,45], the importance of chloroplast signals in ICS1 induction needs to be clarified by further study.

ICS1 is the key enzyme of SA biosynthesis, which is required for both local and systemic acquired resistance, while SA synthesized through this pathway seems to potentiate plant cell death [46,47,48,49,50]. It is well known that SA induces cell death in plants as a defense response [51,52]. We observed the H_2_O_2_ production in the chloroplast of Dex-treated i-hpHSP90C plants (Figure 5) and the upregulation of ROS-responsive genes (Figure 4, Supplementary Table S6). ROS produced in the chloroplast (Figure 6) can induce SA biosynthesis most likely through ICS1 gene activation [49,53,54]. Taken together, the cell death we observed in the chlorosis model plants (Figure 5) is suggested to be first triggered by the ROS production and then activated through the SA-ROS self-amplification loop [55]. Although with the least plant growth, our preliminary experiment of plant culture in the dark suggests that chlorosis development requires light-dependent ROS production (Supplementary Figure S4). Studies have reported that light-dependent ROS production in the chloroplast leads to HR-like cell death and antiviral resistance [56,57,58,59]. However, the significance of SA in cell death in the present model system needs to be studied further.

In addition to defense genes, we found that ER stress response genes were upregulated in the chlorosis model (Table 1 and Supplementary Table S9). Although physical interaction between chloroplasts and ER has been demonstrated, no protein transport was confirmed between these organelles [60,61,62]. Therefore, it is unlikely that loss of protein quality control in the chloroplast could directly induce the unfolded protein response (UPR). The indirect induction of UPR by the impaired HPS90C supply would involve either retrograde signaling or stress hormone response. A retrograde signal molecule, methylerythritol cyclodiphosphate (MEcPP), has been shown to induce UPR in ER [63,64], and SA has been shown to activate the major IRE1-bZIP60 pathway of UPR [24,65]. Further study of the mode of induction of UPR would provide us with insight into its role in chlorosis development.

**Figure 7:**
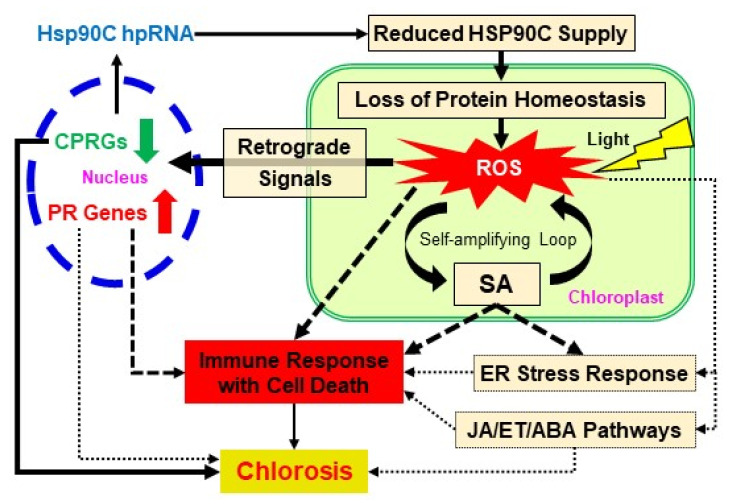
A schematic model for the molecular mechanism of chlorosis. Inducible silencing of the HSP90C gene causes a reduction of HSP90C levels in the chloroplast, which causes the loss of protein homeostasis in the chloroplast. The disruption of protein homeostasis leads chloroplasts to produce ROS in a light-dependent manner. The effects of ROS and SA are enhanced by a self-amplifying loop. ROS serves as the chloroplast retrograde signal and activates other retrograde signaling pathways, leading to the upregulation of pathogenesis-related (PR) genes and the downregulation of chloroplast- and photosynthesis-related genes (CPRGs). Reduced supply of HSP90C in the chloroplast induces the activation of the ER stress response and the upregulation of JA/ET/ABA pathway genes, likely through SA, ROS, or other signaling pathways. ROS, SA, the activation of PR genes, and other stress responses including JA/ET/ABA pathways could stimulate the cell death pathway. The downregulation of CPRGs may be the primary cause of chlorosis, and ROS-mediated triggering of HR-like cell death in the chlorotic tissues could have a role in the development of chlorosis. Solid line arrows indicate the steps with experimental support, whereas broken line arrows are hypothetical.

We summarize the molecular events discussed above that lead to the development of chlorosis after the induced silencing of the HSP90C gene in Figure 7. RNA-seq data have shown the downregulation of nuclear genes under the GO term “protein localization to the chloroplast” (GO:0072598) (Supplementary Table S10). Reduced HSP90C supply can impair the protein homeostasis in the chloroplast which can hamper normal chloroplast function, thus leading to the production of ROS in the chloroplast. ROS can induce SA production, developing a self-amplifying loop. ROS can also activate the chloroplast retrograde signaling, which results in the upregulation of stress-responsive genes, including pathogenesis-related (PR) genes, and the downregulation of CPRGs in the nucleus, which is most likely to be the primary cause of chlorosis. In addition, the HR-like cell death pathway would be activated by ROS, the SA pathway, other stress hormone pathways, and/or UPR. It is well known that chlorosis induced by different cues leads to the death of plant tissue. Therefore, the activation of the cell death response could have a role in chlorosis induction or acceleration of the processes leading to visible chlorosis. Although the importance of cell death induction in chlorosis remains to established, the present study sheds light on the importance of prior activation of the cell death pathway in chlorosis development.

In the case of a virus or viroid infection, the activation of the immune pathway must be unfavorable to the pathogens. Therefore, it is an acceptable idea that pathogens have some way to inhibit the defense response activated by the chloroplast stress. Indeed, PLMVd and some other viruses such as CMV induce clear bleaching-type chlorosis, in which cell death is unlikely [2,7]. Although we have not tested the cell death response in PLMVd-infected plants, the cell death we found in this study could be a plant response hidden by pathogens’ counter-defense. It is notable that PLMVd infection reportedly produces siRNAs to several other host genes, including NB-LRR-type disease resistance genes, in addition to those to HSP90C [7,66]. The analysis of pathogens’ counter-defense strategies would pave the way for a better understanding of mechanisms underlying the development of virus disease symptoms.

## 4. Materials and Methods

### 4.1. Plant Materials

Transgenic tobacco lines, i-hpHSP90C 6-1 (H-6) and i-hpHSP90C 4–5 (H-4), which express hairpin RNA (hpRNA) corresponding to the HSP90C-specific regions under the control of a dexamethasone-inducible promoter, were described previously [10]. These inducible HSP90C silencing tobacco lines and non-transformant tobacco (*Nicotiana tabacum* cv. Petit Havana SR1) were used in this study. Plants were cultured in a plug tray containing commercial soil mix (Supermix A, Sakata Seeds, Yokohama, Japan) for one week at 25 °C under 16/8 h light/dark cycle condition with an irradiation dose of about 60 μM m^−2^ s^−1^. The seedlings were transferred and grown for additional 2 weeks in a plastic pot (6.0 cm in diameter) containing the mixture (1:1) of vermiculite and Supermix A, with watering every other day with 1000-times diluted Hyponex 6-10-5 (Hyponex Japan, Osaka, Japan) solution twice a week. To induce the transgene expression, 3-week-old control and transgenic tobacco plants were sprayed with freshly diluted 50 µM dexamethasone (Dex) solution containing 0.01% (v/v) Tween-20 using a spray bottle. Control plants were mock-treated by spraying with 0.5% ethanol solution containing 0.01% (v/v) Tween-20.

### 4.2. Extraction and Sequencing of RNA

The RNA was extracted and treated with RNase-free DNase as described previously [32]. For RNA-seq analysis, RNA was extracted from six individual plants from each of the Dex-treated and untreated, control, and H-4 (i-hpHSP90C 4–5) transgenic plant groups at 24 h post-Dex-treatment. Three samples from each plant/treatment group were selected by the integrity of RNA assessed using 2100 Bioanalyzer with RNA-nano chip (Agilent, Tokyo, Japan). Libraries were constructed using poly-A RNA and the TruSeq RNA Sample Preparation v2 kit (Illumina, Tokyo, Japan) according to the manufacturer’s protocol. Hiseq 2500 was used to conduct 100-bp single-end sequencing at Nodai Genome Research Center.

### 4.3. RNA-seq Data Analysis

Raw reads were obtained using bcl2fastq2 (Illumina) with the adaptor sequences removed. The read data were further trimmed using fastq_quality_trimmer with the quality cutoff at 28 and length cutoff at 80. The clean read data were uploaded to the local Galaxy platform [67] and analyzed therein using Salmon [68] with Ntab-TN90-AYMY-SS_NGS.mrna.annot.fna reference transcriptome [69] (downloaded from Sol Genomics Network; https://solgenomics.net/organism/Nicotiana_tabacum/genome) for transcript abundance estimation and expression value computation. We prepared a Transcript ID-AGI code table (Supplementary Table S1) from the fasta file above using a series of UNIX commands to have the expression values with AGI codes because the original gene IDs in the accompanying gff3 file were not compatible with our GO enrichment analysis. Thus, Salmon gave the transcripts per million (TPM) value to each of the transcripts with AGI code annotation. Of 189,413 mRNA transcripts in tobacco reference transcriptome, 121,268 transcripts (64.02%) were annotated with AGI codes, while 68,145 transcripts (35.98%) were omitted from the analysis (Supplementary Table S1).

The detection of DEGs was performed in the Galaxy platform using DESeq2, which provides differential expression analysis methods using negative binomial generalized linear models [70,71]. The ‘Gene Quantification’ files from Salmon analysis were analyzed using DESeq2 with the Transcript ID-AGI code table (Supplementary Table S1) to give regularized log transformation of raw read count data. The ‘plotPCA’ function implemented in the DESeq2 package was used to visualize the sample-to-sample distances.

DESeq2 was run in different groupings: Dex-treated H-4 vs. control H-4, Dex-treated H-4 vs. Dex-treated SR1, and Dex-treated H-4 vs. Control SR1. Then, Venn diagrams showing the DEGs in various combinations were prepared using the web program Venny 2.1 (http://bioinfogp.cnb.csic.es/tools/venny/index.html). The differentially expressed genes were considered significant under the following criteria: corrected p-value (P(adj)) less than 0.05 and Log2(FC) values above 1 or below –1. For functional annotation of DEGs with AGI codes, we performed the GO enrichment analysis using the PANTHER (Protein ANalysis THrough Evolutionary Relationships) Classification System (http://www.pantherdb.org/) [72], which implements one-sided Fisher’s exact test with the multiple-testing correction method being set to FDR. GOs with FDR below 0.05 were considered significant. The heatmap for selected upregulated genes was drawn using ggplot2 function in the heatmap2 program in the Galaxy platform. The relative normalized count data calculated in the Excel program (Microsoft) were used instead of the raw normalized count data from DESeq2 because the latter were not accepted by the program. The expression levels of particular genes in the RNA-seq analysis were compared using the relative normalized count data with statistical analysis using DESeq2.

### 4.4. Quantitative RT-PCR

Total RNA was extracted using the ISOSPIN Plant RNA Kit (Nippon Gene, Tokyo, Japan) for qRT-PCR with more plant samples. The cDNA was synthesized using the M-MLV RTase (New England Biolabs Japan, Tokyo, Japan) and subjected to real-time qPCR using StepOnePlus real-time PCR system (Applied Biosystems, Thermo Fisher Scientific, Tokyo, Japan) with KAPA SYBR FAST qPCR master mix (Kapa Biosystems, via Nippon Genetics, Tokyo, Japan). The qPCR conditions were as follows: initial holding at 95 °C for 20 s, 40 cycles of 95 °C for 3 s, 60 °C for 30 s, followed by 95 °C for 15 s, 60 °C for 1 min, 95 °C for 15 s. A melting curve was generated to confirm the specificity of the reactions. Each sample was tested in triplicate. The relative expression level of target genes was calculated by comparative CT (ΔΔCT) method using EF1α as the internal reference and a common standard sample (a mixture of RNAs from untreated SR1) for the normalization among assay plates. The primers used for qRT-PCR analysis are listed in Supplementary Table S2.

### 4.5. Determination of Cell Death

Cell death was measured using the Trypan Blue Assay as described previously [73] with slight modifications. Three-week-old transgenic and control plants were Dex- or solvent-treated, kept for seven days, and observed for the phenotypic changes. The agroinfiltration–mediated transient expression of *tomato bushy stunt virus* (TBSV) P19 [74] was analyzed 2 days post-infiltration as a positive control for cell death [75]. Leaf disks of 6 mm in diameter were heated for three minutes in trypan blue solution, cooled to room temperature, decolorized using chloral hydrate solution, and mounted in 50% glycerol for microscopy analysis. For the quantitative determination of cell death, electrolyte leakage assay was conducted as described previously [76] with slight modifications. Eight leaf disks from each plant were floated in 9 mL sterilized distilled water (SDW) and kept for 24 h under dark conditions. The conductance of the leaked electrolyte in SDW was measured using a conductivity meter (LAQUAtwin-EC-11, HORIBA Scientific, Japan). After the measurement, leaf disks and SDW were recombined and boiled for 10 min, after which the total electrolyte conductivity was measured. The cell death was evaluated with the relative conductivity, namely the ratio of leaked to total electrolyte conductivity, in three triplicate experiments. Statistical analyses (using Tukey’s honestly significant difference (HSD) test were performed using SPSS (Version 17) and Microsoft Office Excel 2016.

### 4.6. DAB (3,3’-Diaminobenzidine) Staining for Hydrogen Peroxide Detection

For in situ detection of hydrogen peroxide (H_2_O_2_), we performed 3,3’-diaminobenzidine (DAB) staining as described previously [77] with slight modifications. Three-week-old transgenic and control plants were Dex- or solvent-treated and further grown for 24 h. Leaf disks of 6 mm in diameter were vacuum-infiltrated with 1 mg/mL DAB solution containing 0.05% Tween-20 in 50 mL conical tubes, kept on wet filter papers in Petri dishes, and incubated under light (70–100 µmol m^−2^ s^−1^ for 30 min for test plants and 250–300 µmol m^−2^ s^−1^ for 60 min for positive control plants) and for an additional 3.5 h in the dark. The leaf disks were decolorized by boiling in 99.5% ethanol for 5–10 min and observed.

## Figures and Tables

**Table 1:** Selected GO terms (biological process) for upregulated genes in i-hpHSP90C transgenic line after Dex treatment.

GO Terms	Total	DEGs	Fold Enrich.	FDR
**Immune system process**	372	101	2.73	<0.01
Innate immune response	300	83	2.78	<0.01
**Response to stimulus**	5327	880	1.66	<0.01
Response to stress	3079	547	1.79	<0.01
Response to osmotic stress	545	105	1.94	<0.01
Response to salt stress	469	90	1.93	<0.01
Defense response	1005	231	2.31	<0.01
Response to wounding	207	59	2.86	<0.01
Response to oxidative stress	392	83	2.13	<0.01
Response to hormone	1360	235	1.74	<0.01
Response to SA	206	41	2	<0.01
Response to JA	217	46	2.13	<0.01
JA-mediated signaling pathway	67	23	3.45	<0.01
Response to ABA	524	103	1.98	<0.01
Response to ER stress	97	37	3.83	<0.01
ERAD pathway	60	17	2.85	0.01
ER unfolded protein response	36	13	3.63	0.01
**Metabolic process**	7731	992	1.29	<0.01
Cellular respiration	80	21	2.64	0.01
Protein glycosylation	104	24	2.32	0.01
Protein autophosphorylation	183	46	2.53	<0.01
JA metabolic process	50	14	2.81	0.03
JA biosynthetic process	26	10	3.87	0.02
Regulation of SA metabolic process	20	10	5.02	0.01
Regulation of SA biosynthetic process	12	8	6.7	0.01
**Cellular process**	10,043	1299	1.3	<0.01
Cell death	112	32	2.87	<0.01
Programmed cell death	91	27	2.98	<0.01
Plant-type hypersensitive response	57	25	4.41	<0.01
Regulation of cell death	79	22	2.8	<0.01
Negative regulation of cell death	33	13	3.96	<0.01
**Plant organ development**	946	141	1.5	<0.01
Root morphogenesis	253	43	1.71	0.04
Leaf senescence	103	29	2.83	<0.01

**Table 2:** Selected GO terms (biological process) for downregulated genes in i-hpHSP90C transgenic line after Dex treatment.

GO Terms	Total	DEGs	Fold Enrich.	FDR
**Metabolic process**	7731	1278	1.3	<0.01
Carbohydrate metabolic process	668	146	1.72	<0.01
Lipid metabolic process	728	175	1.89	<0.01
Cellular AA* metabolic process	333	85	2.01	<0.01
Photosynthesis	177	102	4.54	<0.01
Cofactor metabolic process	416	129	2.44	<0.01
Pigment metabolic process	123	48	3.07	<0.01
Vitamin metabolic process	77	31	3.17	<0.01
**Cellular process**	10,043	1592	1.25	<0.01
Cell wall organization	279	60	1.69	0.01
Cell cycle	486	106	1.72	<0.01
Cellular homeostasis	311	62	1.57	0.04
Cellular component organization	2503	454	1.43	<0.01
Plastid organization	298	136	3.6	<0.01
**Response to stimulus**	5327	903	1.34	<0.01
Response to osmotic stress	545	103	1.49	0.01
Response to oxidative stress	392	87	1.75	<0.01
Response to salt stress	469	91	1.53	0.01
Response to auxin	310	72	1.83	<0.01
Response to light stimulus	680	198	2.29	<0.01
**Rhythmic process**	122	43	2.78	<0.01
Circadian rhythm	109	42	3.04	<0.01

* AA = Amino Acid.

## Supplementary Materials

Supplementary materials can be found at https://www.mdpi.com/1422-0067/21/12/4202/s1. Supplementary Figure S1: Comparison between the expression of HSP90C gene and selected DEGs in non-transformed control SR1 and i-hpHSP90C line 6 plants. Supplementary Figure S2: (A–C) MA plot showing the overview of the DEGs in two-group comparison (i.e., Dex treated and untreated). Supplementary Figure S3: Heat maps showing the oxidative, osmotic, and salt-stress-responsive genes. Supplementary Figure S4: Lack of chlorosis response in dark-grown i-hpHSP0C (6-1) plants. Supplementary Table S1: Transcript ID and Gene ID mapping file. Supplementary Table S2: Primers used for qRT-PCR analysis in the present study. Supplementary Table S3: Summary of RNA-Seq data and mapping of the clean reads to *N. tabacum* TN90 reference transcriptome. Supplementary Table S4: List of upregulated DEGs in chlorosis model plants (commonly upregulated DEGs in all three comparisons). Supplementary Table S5: List of downregulated DEGs in chlorosis model plants (commonly downregulated DEGs in all three comparisons). Supplementary Table S6: List of GO terms (biological process) enriched with upregulated DEGs of chlorosis model plants. Supplementary Table S7: List of GO terms (biological process) enriched with downregulated DEGs of chlorosis model plants. Supplementary Table S8: Expression levels of RS-related transcription factor genes.
