# Correction: Islam, S., *et al.* Impaired Expression of Chloroplast HSP90C Chaperone Activates Plant Defense Responses with a Possible Link to a Disease-Symptom-Like Phenotype. *International Journal of Molecular Science* 2020, *21*, 4202

**DOI:** 10.3390/ijms21228461

**Published:** 2020-11-11

**Authors:** Shaikhul Islam, Sachin Ashok Bhor, Keisuke Tanaka, Hikaru Sakamoto, Takashi Yaeno, Hidetaka Kaya, Kappei Kobayashi

**Affiliations:** 1 The United Graduate School of Agricultural Sciences, Ehime University, Matsuyama, Ehime 790-8566, Japan; islamshaikhul2014@outlook.com (S.I.); bhor.sach@gmail.com (S.A.B.); yaeno@agr.ehime-u.ac.jp (T.Y.); kaya.hidetaka.hu@ehime-u.ac.jp (H.K.); 2 NODAI Genome Research Center, Tokyo University of Agriculture, Setagaya, Tokyo 156-8502, Japan; kt205453@nodai.ac.jp; 3 Faculty of Bio-Industry, Tokyo University of Agriculture, Abashiri, Hokkaido 099-2493, Japan; h3sakamo@nodai.ac.jp; 4 Graduate School of Agriculture, Ehime University, Matsuyama, Ehime 790-8566, Japan; 5 Research Unit for Citromics, Ehime University, Matsuyama, Ehime 790-8566, Japan

The authors wish to make the following corrections to this paper [1]:

There was a misunderstanding in the annotation of a single gene. The gene, ICS1, has been mentioned several times in the main text and graph labels, but the gene should have been annotated to be SARD1, a regulator of ICS1. Although this mistake does not affect the conclusion of our paper, it is very misleading to any readers of the article. Therefore, the authors would like to publish this correction. The correction includes a change in the labels for the horizontal axis in Figure 1c and Supplementary Figure S1; several changes in the main text in the results, the discussion, and the materials and methods sections; and changes in Supplementary Table S2 and the entirety of Supplementary Tables S4, S5, S8, S9, and S10, in which relationships within the columns had been lost during sorting.

The authors would like to apologize for any inconvenience caused to the readers by these changes.
